# Metachronic Breast and Cerebellar Neoplasm in a Young Patient

**DOI:** 10.1055/s-0040-1701456

**Published:** 2020-02

**Authors:** Jéssika de Oliveira Nascimento, Lara Caroline Anastacio Haro, Rafael da Silva Sá, Rebeca Espelho Storch

**Affiliations:** 1 Universidade do Oeste Paulista, Presidente Prudente, SP, Brazil

**Keywords:** medulloblastoma desmoplastic, breast cancer, carcinoma mucinous mammary, Li-Fraumeni syndrome, meduloblastoma desmoplásico, câncer de mama, carcinoma mucinoso mamário, síndrome de Li-Fraumeni

## Abstract

Several factors trigger the development of genetic mutations that are responsible for causing a neoplasm. Medulloblastoma is a malignant and invasive cerebellar neoplasm, that affects children and young adults. Mucinous carcinoma is a special type of breast cancer. Being a special atypical subtype of invasive carcinoma, it most frequently affects women of advanced age and represents 1 to 7% of all breast cancers. The reported case aims to show the rarity of the occurrence of desmoplastic medulloblastoma and mammary mucinous carcinoma in a young patient in a short period of time, in different sites, without direct anatomical attachment and without occurrence of metastasis. Initially, this patient had a desmoplastic medulloblastoma and was treated with lumpectomy and radiotherapy. After 13 months, the patient was diagnosed with a mucinous breast carcinoma, underwent mastectomy, adjuvant chemotherapy and is currently undergoing endocrinotherapy. We conclude, based on the metachronous characteristic of the neoplasia and clinical characteristics, that the patient is likely to have Li-Fraumeni syndrome, an autosomal dominant disease with mutation of the TP53 gene, which is the the main involved. Because the patient does not present all the characteristics of the phenotype of the syndrome, she can thus be classified as having Li-Fraumeni variant or Li-Fraumeni-like syndrome.

## Resumo

Diversos fatores desencadeiam o desenvolvimento de mutações genéticas que são responsáveis por originar uma neoplasia. O meduloblastoma é uma neoplasia cerebelar maligna e invasiva que acomete crianças e adultos jovens. O carcinoma mucinoso é um tipo de câncer de mama especial por ser um subtipo atípico de carcinoma invasivo, que acomete com maior frequência mulheres de idade avançada e representa entre 1 a 7% do total de neoplasias mamárias. O caso relatado tem como objetivo mostrar a raridade da ocorrência do meduloblastoma desmoplásico e carcinoma mucinoso mamário em uma paciente jovem em um curto período de tempo, em diferentes sítios sem ligação anatômica direta e sem ocorrência de metástase. Inicialmente, esta paciente possuía um meduloblastoma desmoplásico e foi tratada com tumorectomia e radioterapia. Após 13 meses, a paciente foi diagnosticada com carcinoma mucinoso de mama, sendo submetida a mastectomia, quimioterapia adjuvante e atualmente está sendo tratada com endocrinoterapia. Concluímos, com base na característica metacrônica da neoplasia e características clínicas, que a paciente apresenta a síndrome de Li-Fraumeni, doença autossômica dominante com mutação do gene TP53, que é o principal gene envolvido nesta síndrome. Por não apresentar as características completas do fenótipo da síndrome, a paciente pode assim ser classificada como portadora de uma variante da síndorme de Li-Fraumeni ou síndrome do tipo Li-Fraumeni.

## Introduction

Neoplasias originate from the union of several factors: genetic, psychological, environmental, habits, and addictions. The association of these factors leads the individual to develop mutations in the genes, generating a rapid cell proliferation and, consequently, forming a tumor mass.^1^ Neoplastic cells have mutations in the mechanisms of cell cycle control, especially the structures involved in DNA repair, and in the mechanism of apoptosis, thus occurring to the proliferation of a damaged cell. The stimulus for this proliferation in most neoplasias is still unknown.^2^ Cancer can be considered an isolated genetic disease, being characterized by hereditary cancer syndromes, which created the dominant autosomal disorders (50% of the chance of transmission of genes related to one of the offspring syndromes in each pregnancy), that make malignant neoplasms prevalent in individuals from the same family. Some characteristics are associated with hereditary cancer, such as early age at diagnosis, more than one neoplasm in the same individual, several members of the same family presenting the same or related neoplasms, and multiple generations affected.^3^ ^4^

Patients with more than one type of neoplasia can be classified into two types: synchronous tumor or metachronous tumor. Metachronic tumors are found in the follow-up of patients already submitted to surgical resection, evidently excluding the possibility of undiagnosed lesion at the time of diagnosis or recurrence of the operated tumor.^5^ The primary synchronic tumor is defined when it occurs simultaneously with the index tumor (the first tumor identified in the patient) or when it is identified within 6 months after discovery of the index tumor. When this period exceeds 6 months, the case is considered as a primary metachronous tumor.^6^ Multiple primary tumors present an incidence of 2 to 17%, and some of the risk factors are: changes in germ line, exposure to therapies, occupational risks, and lifestyle influences.^7^

Medulloblastoma is a malignant and invasive cerebellar neoplasm that manifests in children and young adults with dissemination through the cerebrospinal fluid.^8^ ^9^ Among adults, 80% occur between 21 and 40 years of age. According to the World Health Organization (Louis et al, 2007),^10^ medulloblastomas are classified histologically into 5 types: desmoplastic/nodular (pale islands, highly proliferative cells, nodules with reduced cellularity), extensive nodular, classic, large cell, and anaplastic.^11^ ^12^

The nodular/desmoplastic medulloblastoma has characteristics that distinguish it from typical tumors, since it is usually well circumscribed, contains a large amount of reticulin fibers and, when completely removed, has a better prognosis.^13^ It is mainly located in the cerebellar hemisphere but may also be located in the cerebellar vertebra.^14^ Some authors consider less aggressive histological alterations due to the lower occurrence of necrosis, lower mitotic index, and nuclear alterations are classified as mild when compared with the group of classical medulloblastomas.^13^

The clinical picture is insidious and progressive, making the patient complain of lumbar or radicular pain, seizures, cranial hypertension, and focal medullary or encephalic symptoms due to neoplastic implants. Headache appears as a predominant symptom in adults with this type of tumor. Imbalance, gait disturbance, dizziness, and/or nausea and vomiting are common. Other symptoms rarely referred to in adults are diplopia, loss of limb strength, tinnitus, deafness, photophobia, secondary amenorrhea, mental confusion, syncope, visual blurring or blindness, drowsiness, nuchal and dorsal pain, dysarthria, dysphagia, maxillary, urinary incontinence, weight loss, and partial or generalized epileptic seizures.^15^ Surgery is the most important aspect of medulloblastoma treatment, and tumor resection is ideal.^12^

Breast cancer is the second most common type of cancer in the general population, just behind basal -cell skin cancer. The National Cancer Institute estimates that 59,700 new cases of breast cancer will be expected in 2018, corresponding to 29% of female cancers, in Brazil.^16^ Mucinous cancer, also known as colloid carcinoma of the breast, is considered an atypical subtype of invasive carcinoma; it affects more frequently women of advanced age and represents 1 to 7% of the total of mammary neoplasias^9^. Mucins are complex carbohydrates released by connective tissue cells and mainly by specialized epithelial cells. They represent the main constituent of mucus and play a protective role in tissues composed of epithelial cells. In addition to the protection factor, they are involved in processes of epithelial differentiation, cellular signaling and cellular adhesion modulation.^17^ Within the mucinous tumors, there is differentiation between carcinoma of pure form and carcinoma of mixed form. The carcinoma of pure form shows the mucinous arrangement in practically the entire tumoral extent, while the carcinoma of mixed form has a greater extension of neoplastic cells not surrounded by mucin. The higher the amount of mucin the better the prognosis.^9^

The objective of the present work is to identify and analyze the relationship between a nodular/desmoplastic medulloblastoma (grade IV) and the subsequent development of intermediate grade mammary mucinous carcinoma in a young patient, considering that there was no metastasis involved between the distinct anatomical periods and locations and that there was no direct correlation.

The case report was approved by the local Research Ethics Committee under CAAE 04155318.6.0000.5515 and CEP Opinion Number 3,193,241.

## Case Description

**Fig. 1:**
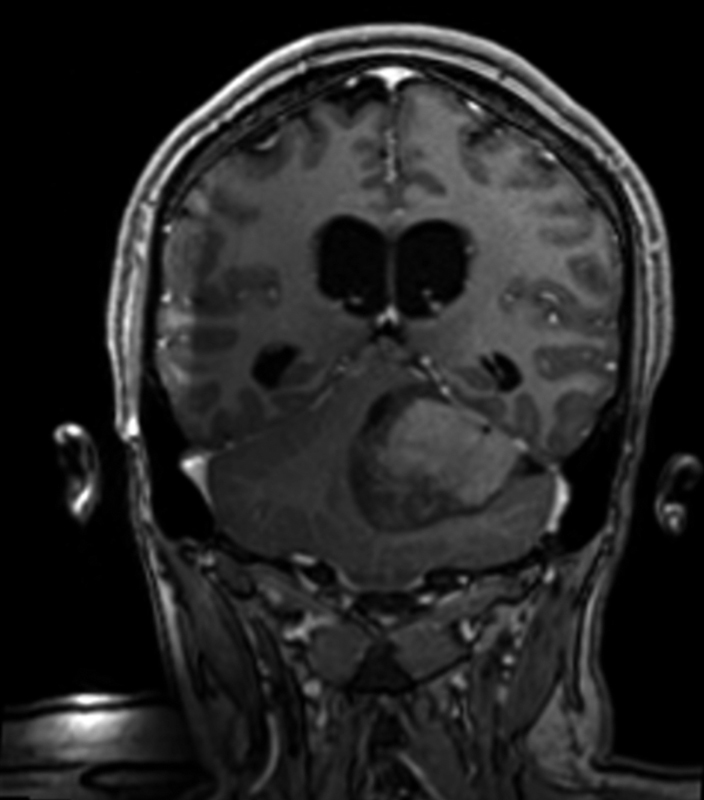
Magnetic resonance imaging of the skull shows expansive lesion in the posterior fossa with epicenter in the cerebellar vermis/IV ventricle, solid and cystic.

**Fig. 2:**
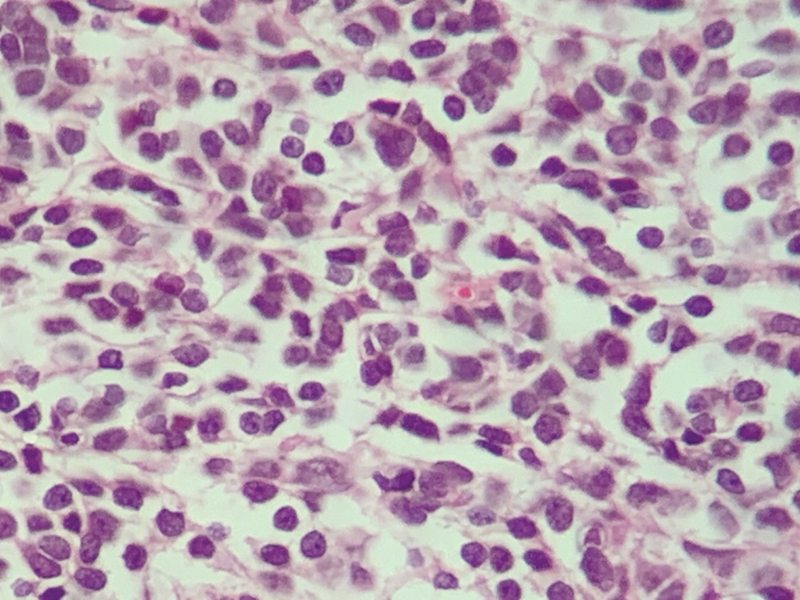
Cerebellar tumor slide (600x magnification) showing oval cells with hyperchromatic nuclei, well-marked nuclear membranes, and scarce cytoplasm with pseudo-rosettes.

The patient is female, 29 years old, Caucasian, single, hairdresser, natural and from Sandovalina, SP. Patient denies family history of neoplasms. She consulted with a neurologist due to a complaint of headache, vertigo, blurred vision, ataxia, without episodes of syncope or lipothymia. Magnetic resonance imaging (MRI) of the skull revealed an expansive lesion in the posterior fossa with epicenter in the cerebellar/IV ventricle, which was a cystic and solid, causing erasure of the IV ventricle and a mild/moderate supraventricular hydrocephalus. After anatomopathological examination (macroscopy reveals an irregular portion of tissue, measuring 2.5 × 2 cm, with a smooth grayish surface. At cuts, the surface is whitish and firm-elastic in consistency, also accompanied by several irregular tissue fragments, measuring 2 × 1 cm together and of light brown color and firm consistency (Fig. 1). On microscopy, we observed the presence of malignant neoplasia consisting of oval cells with hyperchromatic nuclei, well-marked nuclear membranes, and scarce cytoplasm with pseudo-rosettes; the cells exhibited several atypical figures of mitoses, the vascular network consisted of thin capillary vessels, and there was presence of irregular areas of necrosis (Fig. 2). In the immunohistochemistry (GCDFP-15 antigen negative, K_i_-67 antigen indicating high cell proliferation index and synaptophysin antigen diffusely positive) examination, there was compatibility with nodular/desmoplastic medulloblastoma. The patient was submitted to lumpectomy with adjuvant radiotherapy, which was started 4 months after the surgical procedure (29 sessions).

Thirteen months after the neurological lesion, the patient was referred to the division of mastology due to palpable lump in the left breast. The bilateral mammography report, performed 3 months previously, presented microcalcifications with discrete grouping in the upper left quadrant and an image with nodular effect in the upper left quadrant to the left of the mean density and partially defined contours, being classified as category 3 of the breast, imaging, reporting and data system (BI-RADS). Physical examination showed a hardened nodule in the periareolar left lateral quadrant measuring 3 cm, free axillae and absence of papillary effusion (clinical staging: T2N0M0 - IIA). The histopathological pattern (multifocal lesion, being larger than 2.5 cm and smaller than 0.9 cm in diameter, all whitish colors, finely grainy surface, firm consistency and unctuous appearance) associated with the immunohistochemical profile (CerbB-2 oncoprotein antigen negative [score 1], K_i_-67 antigen moderate cell proliferation index, estrogen receptor antigen positive in 90% of neoplastic cells, progesterone receptor antigen positive in 50% of neoplastic cells) was compatible with mammary mucinous carcinoma, intermediate grade, infiltrative, with approximate molecular classification of luminal A. Due to its location, tumor size and tumor-breast ratio, the patient underwent a mastectomy associated with the left sentinel lymph node biopsy (benign to biopsy) and adjuvant chemotherapy (4 cycles of doxorubicin 108 mg and cyclophosphamide 1,080 mg combined with 4 cycles of docetaxel 100 mg / m^2^). Currently, tamoxifen is being used continuously (20 mg/day).

## Discussion

Medulloblastoma has a strong tendency of dissemination along the cerebrospinal pathways. The extraneural or systemic spread is rare; when it occurs, the most common sites are bone marrow, bones, peritoneum, lungs, and liver, respectively.^18^ Therefore, medulloblastoma metastasis to breasts tissue is considered uncommon. However, in 2010, Ternier et al^19^ described the case of a 29-year-old woman who developed a medulloblastoma and later breast carcinoma. The results of the immunohistochemistry established that metastasis had occurred, confirming the primary site.

In the clinical description of the case discussed in the present study, one patient presented breast mucinous lump cancer posterior to the medulloblastoma, and, according to the literature, extraneural dissemination of this tumor is rare. The possibility that this patient was a case of metastasization was ruled out choice of use distinct bonds, having a different immunohistochemistry and showing no relation between the tumors. Consideration should be given to the possibility of the patient having a genetic mutation of important cell division regulating genes, namely: growth promoters, cell growth inhibitors, and genes that regulate the cell cutting program.^20^ Among them, we highlight the cell growth inhibitory genes, also called tumor suppressors, in which the main alteration is in the TP53 gene (encoder of p53 protein—this protein has several antitumor effects, remains genetic, and inhibits angiography through cellular apoptosis). Therefore, with a mutation of this gene, there is no efficient DNA repair, thus contributing to the formation of abnormal cells.^20^ ^21^

Due to the fact that the patient presented tumors in different primary sites, a hypothesis was formed that the patient was a carrier of the Li-Fraumeni syndrome (LFS), an autosomal dominant disease in which the TP53 mutation is the main gene involved.^22^ Li-Fraumeni syndrome relates to the diagnosis of various types of tumors in young patients, multiple primary tumors, and a characteristic pattern of family grouping of a variety of cancers (central tumors including: bone and soft tissue sarcomas, central nervous system tumors, leukemia, adrenocortical carcinoma, and breast cancer). This syndrome differs from other hereditary cancer syndromes because it is not related to a specific type of cancer, but to a broad spectrum of tumors.^23^ Syndrome variants include LFS1, LFS2 and LFS-like (LFSL).^21^

In the South and Southeast regions of Brazil, there is a high frequency of Li-Fraumeni and Li-Fraumeni-like syndromes, because most of the population in these areas is of European origin. The hypothesis of a possible founding mutation, also called the founding effect, was raised, that is, there was a first carrier of the mutation that spread to the populations of the South and Southeast of Brazil. This fact can be explained by the Brazilian colonization. At the end of the 17^th^ century, the southern region received Portuguese immigrants, known as “drovers;” they created routes toward the southeastern region to trade cattle and consumer goods. Thus, although the insertion of a mutant Portuguese allele in the colonization period took place when the population was small, it led to a currently significant prevalence in the population.^22^ ^24^

The patient in this report does not have complete characteristics of the phenotype of the syndrome, and can thus be classified as having Li-Fraumeni variant or Li-Fraumeni-like (individuals with no detectable mutations in the P53 protein of the gene TP53), subclassified by the Chompret criteria, which is characterized by multiple primary tumors, with at least 2 tumors being in the group: sarcoma, a tumor of the central nervous system, breast cancer, or adrenocortical carcinoma, regardless of family history; these should be diagnosed before 36 years of age.^21^ ^22^ Other clinical criteria for diagnosis of the syndrome are briefly described in Table 1.

**Table 1:** Clinical criteria for Li-Fraumeni and Li-Fraumeni-like syndromes

Clinical criteria	Description
Classical Li-Fraumeni	I-sarcoma diagnosed in childhood/young adulthood (≤ 45 years) and II-first-degree relative with any cancer in young adulthood (≤ 45 years) and III-first- or second-degree relative with any cancer diagnosed in young adulthood (≤ 45 years) or sarcoma diagnosed at any age.
Li-Fraumeni-like – criteria of Birch	I-childhood cancer (at any age) or sarcoma, CNS (tumor, or ACC in young adulthood (≤ 45 years) and II-first- or second-degree relative with LFS-spectrum cancer (sarcoma, breast cancer, CNS tumor, ACC, leukemia) at any age and III-first- or second-degree relative with any cancer diagnosed at age < 60 years
Li-Fraumeni-like – criteria of Eeles 1, Eeles 2	I-at least 2 first- or second-degree relatives with LFS-spectrum cancer (sarcoma, breast cancer, CNS tumor, ACC, leukemia, melanoma, prostate cancer, pancreatic cancer) diagnosed at any age II-sarcoma diagnosed at any age and III-at least 2 other tumors diagnosed in one or more first- or second-degree relatives: BC at age < 50 years; CNS tumor, leukemia, ACC, melanoma, prostate cancer, pancreatic cancer at age < 60 years; or sarcoma at any age.
Li- Fraumeni-like – criteria of Chompret	I-diagnosis of sarcoma, CNS tumor, breast cancer, ACC at age < 36 years and II-first- or second-degree relative with any of the above cancers (except BC if proband had BC) or relative with multiple primary tumors at any age or III-multiple primary tumors, including two of the following: sarcoma, CNS tumor, BC, or ACC, with the first tumor diagnosed at age < 36 years regardless of family history; or IV-ACC at any age, regardless of family history.
Li-Fraumeni-like – criteria of Modified Chompret	I-index case with LFS-spectrum cancer (sarcoma, breast cancer, CNS tumor, ACC, leukemia, bronchioloalveolar carcinoma) occurring at age < 46 years and II-a first- or second-degree relative with LFS-spectrum cancer occurring at age < 56 years (except BC if the index case has BC as well), or multiple tumors; or III-index patient with multiple tumors, at least two of which are in the LFS spectrum, the first occurring at age < 46 years; or IV-ACC or choroid plexus carcinoma occurring at any age or BC occurring at age < 36 years without *BRCA1* or *BRCA2* mutations.

Abbreviations: ACC, adrenocortical carcinoma; BC, breast cancer; CNS, central nervous system; LFS, Li-Fraumeni syndrome.

**Source:** Giacomazzi et al.^23^

The most frequent tumors in the TP53 gene mutation are, respectively, breast, soft-tissue sarcomas, adrenocortical, and central nervous system, among others. Up to 30% of women with the syndrome are affected, characterizing a great importance in the diagnosis of breast cancer in women with the syndrome and mutation of the TP53 gene.^22^ It is recommended that these patients are referred to a geneticist, seek a detailed family register, perform a screening for new cancers and clarify the risks. Psychological monitoring is very important as it can cause anxiety or depression.^21^

## Conclusion

Due to the metachronous characteristic of the neoplasia and the clinical presentation displayed by the patient, we concluded that she probably carries Li-Fraumeni syndrome, an autosomal dominant disease. However, the patient of this report does not have complete characteristics of the phenotype of the syndrome, and can thus be classified as having Li-Fraumeni variant or Li-Fraumeni-like syndrome; however, as a consequence of the lack of local resources of the Unified Health System (it does not offer the service of medical genetics), it was not possible to confirm the mutation of the TP53 gene. The positivity of the test would be very useful to the family of this patient, to hereditary investigation and primary and secondary prevention of some neoplasias included in the Li-Fraumeni syndrome group. Unfortunately, the reality is discordant: few medical centers outside the state capitals have the ability to perform genetic mutation tests. In fact, this case report has relevance for the medical community to elucidate a pathology little known among the population.
